# Self-reported snoring is associated with chronic kidney disease in obese but not in normal-weight Chinese adults

**DOI:** 10.1080/0886022X.2021.1915332

**Published:** 2021-04-26

**Authors:** Ziyun Jiang, Jun Qin, Kai Liang, Ruxing Zhao, Fei Yan, Xinguo Hou, Chuan Wang, Li Chen

**Affiliations:** a Department of Endocrinology, Qilu Hospital, Cheeloo College of Medicine, Shandong University, Jinan, China; b Institute of Endocrine and Metabolic Diseases of Shandong University, Jinan, China; c Key Laboratory of Endocrine and Metabolic Diseases, Shandong Province Medicine & Health, Jinan, China; d Jinan Clinical Research Center for Endocrine and Metabolic Diseases, Jinan, China

**Keywords:** Snoring, obesity, normal weight, chronic kidney disease

## Abstract

**Background:**

The relationship between sleeping disorders and chronic kidney disease (CKD) has already been reported. Snoring, a common clinical manifestation of obstructive sleep apnea–hypopnea syndrome, is of clinical value in assessing sleeping disorder severity. However, investigations of the connection between snoring and CKD are limited, especially in normal-weight populations. This study assessed the relationship between snoring frequency and CKD in obese and normal-weight people in China.

**Methods:**

A community-based retrospective cross-sectional study of 3250 participants was performed. Study participants were divided into three groups – the regularly snoring group, occasionally snoring group, and never snoring group – based on their self-reported snoring frequency. CKD was defined as an estimated glomerular filtration rate of less than 60 mL/min/1.73 m^2^. Multiple logistic regression analysis was used to explore the relevance between snoring frequency and CKD prevalence.

**Results:**

The CKD prevalence in obese participants was higher than that in normal-weight participants. Frequent snorers had a higher prevalence of CKD than those who were not frequent snorers in the obese group. Snoring frequency was correlated with CKD prevalence in obese participants independent of age, sex, smoking and drinking status, systolic blood pressure, triglyceride level, high-density lipoprotein, and homeostasis model assessment of insulin resistance (odds ratio: 2.66; 95% CI: 1.36–5.19; *p*=.004), while the same relationships did not exist in normal-weight participants (odds ratio: 0.79; 95% CI: 0.32–1.98; *p*=.614).

**Conclusions:**

Snoring appears to be independently associated with CKD in obese but not in normal-weight Chinese adults.

## Introduction

Chronic kidney disease (CKD) is a comprehensive public health problem with a growing prevalence. Studies have shown that large gains in weight (>10%) and increases in waist circumference (>15%) independently predict incident CKD [1]. Regardless of whether there is a metabolic abnormality, it has been suggested that obesity is associated with an increased risk for CKD progression [1–3]. Specifically, angiotensinogen and leptin produced by adipocytes can alter the renal microcirculation in the early stages of the disease [4].

Meanwhile, obstructive sleep apnea–hypopnea syndrome (OSAHS) is a common condition that affects 45% of obese individuals [5]. Patients with OSAHS are at greater risk for developing CKD with or without metabolic syndromes such as hypertension and diabetes [6]. Possible mechanisms of the interaction between OSAHS and CKD have been discussed, including hypoxia, inflammation, oxidative stress, sympathetic nervous system activation, and activation of the renin–angiotensin–aldosterone system [7]. However, studies on the connection between OSAHS and CKD in normal-weight people are limited in number, possibly because normal-weight people have lower prevalence rates of OSAHS and metabolic syndrome [8], which means that further research in this area is necessary.

Snoring is the most common symptom of OSAHS since up to 94% of patients with OSA display this symptom [9]. On one hand, it is reported that snoring increases the risk for cardiovascular diseases (CVDs) events both in the American and Asian population [10,11], and CVD are risk factors for the morbidity and death of CKD [12]. On the other hand, snoring is an intuitive, convenient, and consistent assessment indicator [13] that can reflect the severity of OSAHS. Therefore, we aimed to study the correlation between snoring frequency and CKD prevalence and investigated the relevance of our findings in both obese and normal-weight participants with the goal of adding to the literature on risk factors for CKD to support earlier intervention when possible.

## Materials and methods

### Data collection

This study is a part of REACTION study [14]. We used a standard questionnaire which is designed strictly to collect information on demographic characteristics and lifestyles of the participants by talking face to face. Only those who knew the details of their snoring frequency were selected. Current smoking status and alcohol drinking status were described as binary variables. Body mass index was calculated as weight (kg) divided by height squared (m^2^). Blood pressure (BP) was surveyed on the left arm three times continuously after the participants sat for at least five minutes, with the average reading of all three records used for analysis. Measurements of fasting blood glucose, triglyceride (TG) level, high-density lipoprotein cholesterol (HDL-C), and creatinine were collected by collecting venous blood after overnight fasting for at least 10 h. The estimated glomerular filtration rate (eGFR) was calculated from the creatinine level using the Chronic Kidney Disease Epidemiology Collaboration (CKD-EPI) formula [15]. All the relevant data are presented in Supplemental Files-data.xlsx.

### Study population

A total of 10,028 adults older than 40 years old in Shandong province were randomly convened from January to April 2012 as described previously [16]. Ineligible study participants were ruled out based on the following criteria: (1) missing data for the diagnosis of obesity and eGFR calculations; (2) those who were not sure about their snoring frequency; (3) previously diagnosed with kidney diseases such as nephritis, nephrotic syndrome, renal fibrosis, and renal failure; (4) previously diagnosed with a hepatic disease such as steatohepatitis, liver cirrhosis, and autoimmune hepatitis; and (5) the presence of any malignant disease. In the end, 3250 participants (including 2041 women) were included in this study for analysis.

### Study design and methods

**Figure 1.**
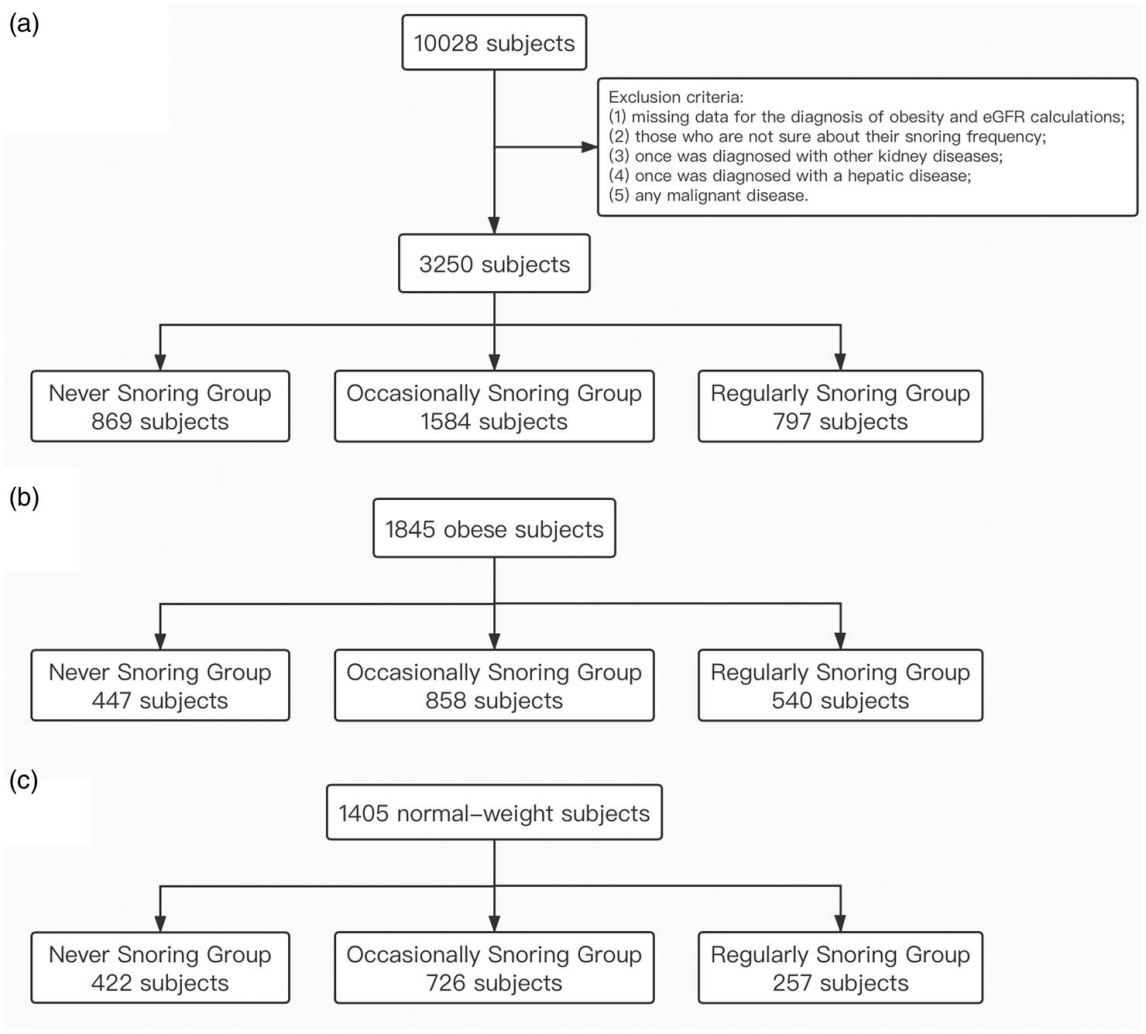
(a) A total of 3,250 participants were divided into three groups based on snoring frequency. (b) A subgroup of 1845 obese participants was stratified according to the same three groups based on snoring frequency. (c) A subgroup of 1405 normal-weight participants was stratified according to the same three groups based on snoring frequency.

The 3250 total study participants were divided into the following three groups based on their self-reported snoring frequency (Figure 1(a)): regularly snoring group (≥3 nights per week), occasionally snoring group (frequency between that of the regularly snoring group and never snoring group), and never snoring group (<1 night per month) [17]. In addition, we separated 3250 study participants into obese individuals and normal-weight individuals according to their waist circumference (obesity defined as ≥90 cm for men and ≥85 cm for women) [18], and each weight group was stratified according to the same three groups mentioned above (Figure 1(b,c)). The CKD-EPI equation [15] was used to estimate the eGFR as 141 × min(Scr/*κ*, 1)*^α^*×max(Scr/*κ*, 1)^−1.209^×0.993^Age^×1.018, where Scr is the serum creatinine level (mg/dL), *κ* is 0.7 for women and 0.9 for men, and *α* is −0.329 for women and −0.411 for men. Multiple logistic regression analysis was used to explore the association between snoring frequency and CKD prevalence.

### Definitions

For this study, CKD [19] was defined as an eGFR of less than 60 mL/min/1.73 m^2^. Serum creatinine was surveyed via the picric acid method using an automatic analyzer (ARCHITECT ci16200 Integrated System; Abbott Laboratories, Chicago, IL) at the standardized laboratory of Ruijin Hospital in Shanghai. Abdominal obesity (central obesity) was defined as a waist circumference ≥90 cm for men and ≥85 cm for women in China according to the Chinese Guidelines for the Prevention and Treatment of Type 2 Diabetes (2019 edition) [18].

### Statistical analysis

Continuous numerical variables with normal distribution were expressed by mean ± standard deviation values, and differences between the three snoring frequency groups were assessed by a one-way analysis of variance test. Separately, continuous numerical variables with abnormal distribution were expressed by median values, and the differences between the three snoring frequency groups were examined using the Kruskal–Wallis *H* test. Categorical variables were expressed by rates, and the differences between the three snoring frequency groups were reviewed using the chi-squared test. The association between snoring frequency and CKD prevalence was tested by multiple logistic regression analysis. The relevant covariates were adjusted as follows: model 1, not adjusted; model 2, adjusted for age and sex; model 3, adjusted for age, sex, smoking status, and drinking status; model 4, adjusted on the basement of model 3 with the addition of systolic BP, TG level, HDL-C, and HOMA-IR value. All data mentioned were analyzed with the Statistical Package for the Social Sciences version 25.0 software program (IBM Corporation, Armonk, NY).

## Results

### Relevance between somatotype, snoring frequency, and CKD prevalence

**Figure 2.**
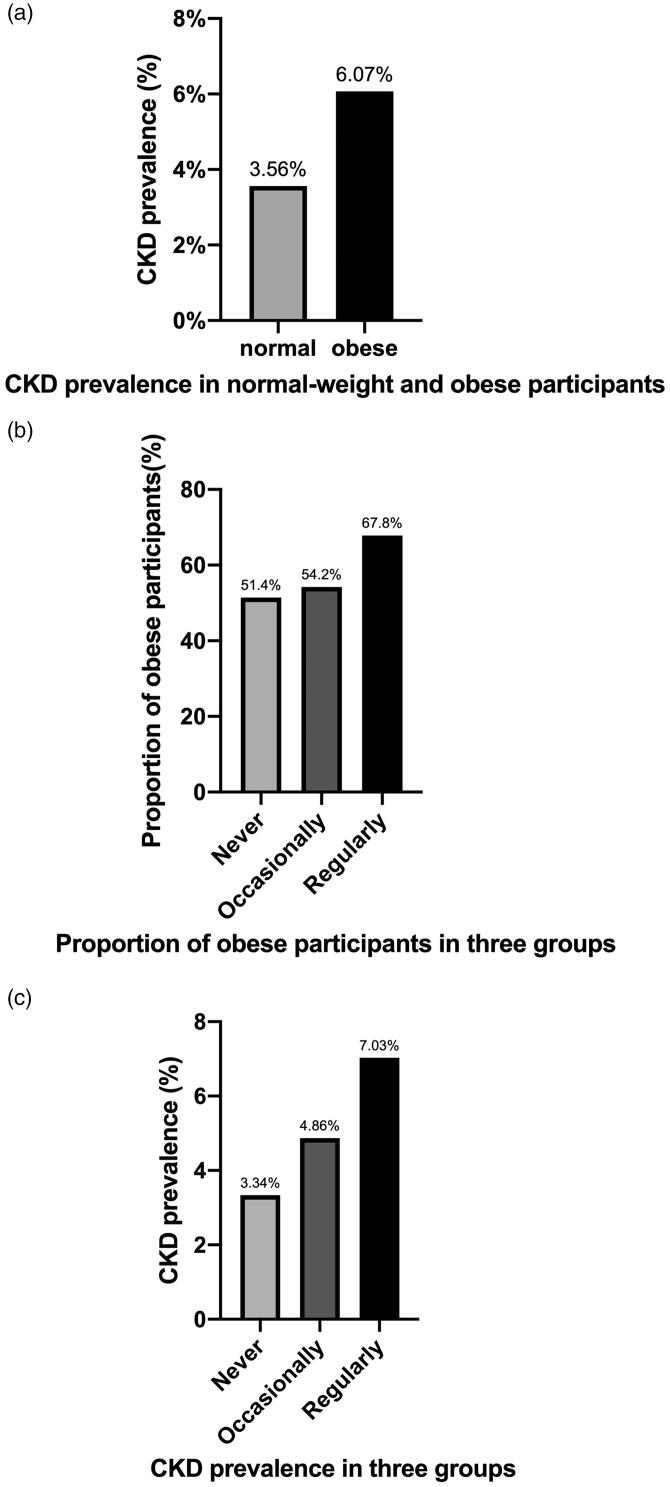
The relationship between somatotype, snoring frequency, and CKD prevalence. (a) The CKD prevalence rates in normal-weight and obese participants were 3.56% (50 CKD patients among 1405 normal-weight participants) and 6.07% (112 CKD patients among 1845 obese participants), respectively. (b) The proportions of obese participants in the never, occasionally, and regularly snoring frequency groups were 51.4% (447 obese participants among 869 participants), 54.2% (858 obese participants among 1584 participants), and 67.8% (540 obese participants among 797 participants), respectively. (c) The CKD prevalence rates among the never, occasionally, and regularly snoring frequency groups were 3.34% (29 CKD patients among 869 participants), 4.86% (29 CKD patients among 1584 participants), and 7.03% (56 CKD patients among 797 participants), respectively.

Table 1 shows the demographic characteristics of whole participants based on snoring frequency. The CKD prevalence of whole participants was 5.0% (162 CKD participants among 3250 participants) (Table 1). Remarkably, the CKD prevalence in obese study participants was higher than that in the normal-weight participants (Figure 2(a)). As mentioned, study participants were divided into three groups based on their self-reported snoring frequency; as the frequency of snoring increased, the proportion of obese participants increased as well (Figure 2(b)). We also found that the more frequent the snoring, the higher the prevalence of CKD (Figure 2(c)), which led to a conclusion that CKD was more prevalent among those who snored more frequently. However, whether it is obesity that affects the relationship between snoring and CKD is still unknown.

**Table 1.** Demographic characteristics of whole participants based on snoring frequency.

Characteristic	Snoring frequency			*p* Value
	Never	Occasionally	Regularly	
*N* (%)	869 (26.7%)	1584 (48.7%)	797 (24.5%)	<.001
Female (%)	609 (70.1%)	1046 (66.0%)	386 (48.4%)	<.001
Age (years)	59.37 ± 9.95	58.99 ± 9.60	60.35 ± 8.82	.003
BMI (kg/m^2^)	25.70 ± 3.25	26.26 ± 3.45	27.05 ± 3.49	<.001
WC (cm)	86.54 ± 9.91	87.37 ± 9.71	91.24 ± 9.86	<.001
SBP (mmHg)	139.15 ± 20.28	140.03 ± 21.23	142.38 ± 19.29	.002
DBP (mmHg)	79.68 ± 11.27	79.89 ± 11.44	81.73 ± 11.47	<.001
FPG (mmol/L)	5.97 ± 1.72	5.99 ± 1.81	6.34 ± 1.91	<.001
Fasting insulin (mIU/L)	7.70 (5.50–11.10)	8.10 (5.90–11.00)	9.00 (6.70–12.50)	<.001
HOMA-IR index	1.92 (1.36–2.68)	2.02 (1.43–2.98)	2.40 (1.67–3.47)	<.001
TG (mmol/L)	1.26 (0.91–1.79)	1.32 (0.94–1.89)	1.46 (1.06–2.08)	<.001
HDL-C (mmol/L)	1.53 ± 0.35	1.49 ± 0.33	1.44 ± 0.30	<.001
eGFR(mL/min/1.73 m^2^)	89.79 ± 13.89	88.73 ± 15.01	84.37 ± 14.51	<.001
Smoking (%)	82 (9.4%)	172 (10.9%)	156 (19.6%)	<.001
Drinking (%)	62 (7.1%)	126 (8.0%)	143 (17.9%)	<.001
CKD (%)	29 (3.3%)	77 (4.9%)	56 (7.0%)	**.002**

BMI: body mass index; WC: waist circumference; SBP: systolic blood pressure; DBP: diastolic blood pressure; FPG: fasting plasma glucose; HOMA-IR: homeostasis model assessment of insulin resistance; TG: triglyceride; HDL-C: high-density lipoprotein cholesterol; eGFR: estimated glomerular filtration rate; CKD: chronic kidney disease.

Data are expressed as mean ± standard deviation, median (interquartile range), or rate (%) values. The bold values represents the prevalence of CKD is different from the three groups based on snoring frequency.

### Characteristics of obese study participants based on snoring frequency

**Figure 3.**
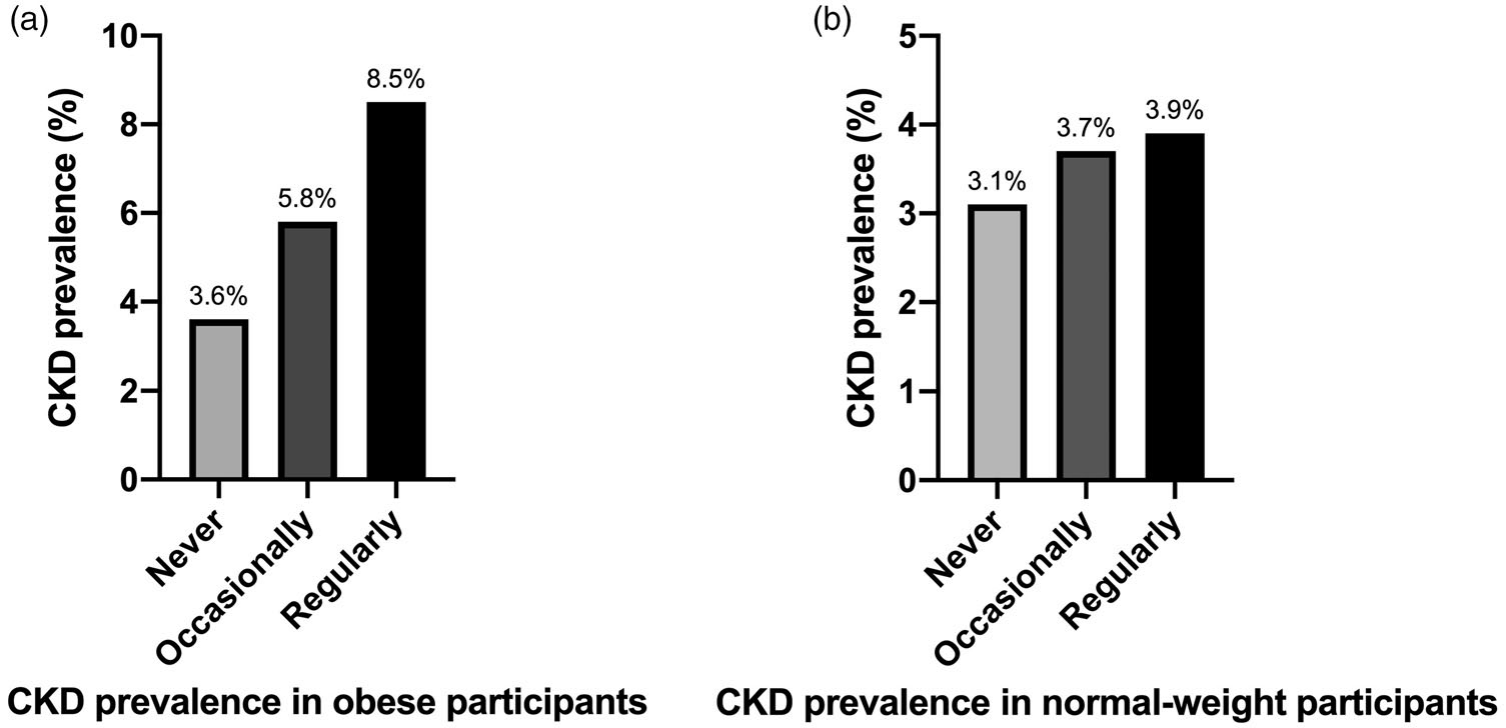
(a) The CKD prevalence among obese participants was statistically different between the three groups according to their snoring frequency (*p*=.005, <.05). (b) Meanwhile, that in normal-weight participants was not (*p*=.811, >.05).

A total of 1845 obese participants (*n* = 1103 women) were divided into three snoring frequency groups. The CKD prevalence among these obese participants was 6.1% (112 CKD participants among 1845 obese participants) (Table 2). As compared with participants in the never snoring group and occasional snoring group, those in the regular snoring group were more likely to be male, smokers, and alcohol drinkers, with higher fasting plasma glucose, higher HOMA-IR, higher TG, and lower HDL-C values. Moreover, CKD prevalence was statistically different between the three snoring frequency groups (*p*<.05) (Figure 3(a)): the more frequently the obese participants snored, the greater the CKD prevalence among them, with the regular snoring group presenting the highest CKD prevalence among the three groups.

**Table 2.** Characteristics of obese and normal-weight study participants based on snoring frequency.

		Snoring frequency			
	Characteristic	Never	Occasionally	Regularly	*p* Value
Obese	*N* (%)	447 (24.2%)	858 (46.5%)	540 (29.3%)	<.001
	Female (%)	298 (66.7%)	539 (62.8%)	266 (49.3%)	<.001
	Age (years)	61.70 ± 9.38	60.26 ± 9.14	60.92 ± 8.74	.023
	BMI (kg/m^2^)	27.34 ± 3.05	27.86 ± 3.12	28.28 ± 3.26	<.001
	WC (cm)	94.13 ± 6.64	94.32 ± 6.43	96.28 ± 7.05	<.001
	SBP (mmHg)	143.07 ± 20.11	143.54 ± 21.17	144.61 ± 19.12	.462
	DBP (mmHg)	81.15 ± 11.11	81.57 ± 11.46	82.85 ± 11.45	.043
	FPG (mmol/L)	6.14 ± 1.56	6.20 ± 1.81	6.44 ± 1.88	.013
	Fasting insulin (mIU/L)	8.60 (6.80–11.50)	9.40 (7.00–12.65)	9.90 (7.40–13.90)	<.001
	HOMA-IR index	2.22 (1.67–3.23)	2.51 (1.75–3.52)	2.75 (1.93–3.86)	<.001
	TG (mmol/L)	1.37 (0.99–2.01)	1.49 (1.07–2.10)	1.54 (1.14–2.26)	<.001
	HDL-C (mmol/L)	1.45 ± 0.31	1.42 ± 0.31	1.40 ± 0.28	.045
	eGFR (mL/min/1.73 m^2^)	86.93 ± 13.47	86.66 ± 14.57	83.03 ± 15.12	<.001
	Smoking (%)	35 (7.8%)	102 (11.9%)	110 (20.4%)	<.001
	Drinking (%)	34 (7.6%)	77 (9.0%)	100 (18.5%)	<.001
	CKD (%)	16 (3.6%)	50 (5.8%)	46 (8.5%)	**.005**
Normal-weight	*N* (%)	422 (30.0%)	726 (51.7%)	257 (18.3%)	<.001
	Female (%)	311(73.7%)	507 (69.8%)	120 (46.7%)	<.001
	Age (years)	56.90 ± 9.96	57.50 ± 9.91	59.16 ± 8.88	.012
	BMI (kg/m^2^)	23.98 ± 2.46	24.37 ± 2.78	24.47 ± 2.49	.024
	WC (cm)	78.52 ± 5.44	79.11 ± 5.60	80.67 ± 5.67	<.001
	SBP (mmHg)	135.02 ± 19.62	135.91 ± 20.51	137.93 ± 18.92	.182
	DBP (mmHg)	78.12 ± 11.22	77.90 ± 11.09	79.38 ± 11.11	.181
	FPG (mmol/L)	5.79 ± 1.85	5.73 ± 1.77	6.13 ± 1.95	.009
	Fasting insulin (mIU/L)	6.50 (4.90–8.90)	6.80 (5.10–9.00)	7.40 (5.30–9.55)	.024
	HOMA-IR index	1.63 (1.17–2.23)	1.63 (1.22–2.29)	1.87 (1.37–2.52)	<.001
	TG (mmol/L)	1.15 (0.84–1.64)	1.15 (0.83–1.60)	1.27 (0.91–1.83)	.014
	HDL-C (mmol/L)	1.60 ± 0.38	1.57 ± 0.33	1.50 ± 0.32	.001
	Smoking (%)	47 (11.1%)	70 (9.6%)	46 (17.9%)	<.001
	Drinking (%)	28 (6.6%)	49 (6.7%)	43 (16.7%)	<.001
	eGFR (mL/min/1.73 m^2^)	92.73 ± 13.76	91.22 ± 15.18	87.11 ± 12.65	<.001
	CKD (%)	13 (3.1%)	27 (3.7%)	10 (3.9%)	**.811**

BMI: body mass index; WC: waist circumference; SBP: systolic blood pressure; DBP: diastolic blood pressure; FPG: fasting plasma glucose; HOMA-IR: homeostasis model assessment of insulin resistance; TG: triglyceride; HDL-C: high-density lipoprotein cholesterol; eGFR: estimated glomerular filtration rate; CKD: chronic kidney disease.

Data are expressed as mean ± standard deviation, median (interquartile range), or rate (%) values. The bold values represents the prevalence of CKD is different from the three groups based on snoring frequency only in obese participants, but not in normal-weight participants.

### Characteristics of the normal-weight participants based on snoring frequency

A total of 1405 normal-weight study participants (*n* = 938 women) were divided into the same three snoring frequency groups. The CKD prevalence among normal-weight participants was found to be 3.6% (50 CKD participants among 1405 normal-weight participants) (Table 2). Study participants in the regular snoring group were more likely to be male, older, smokers, and alcohol drinkers, with higher fasting plasma glucose, higher HOMA-IR, higher TG, and lower HDL-C values, which is similar to the findings in obese participants. However, the difference in the CKD prevalence between the three snoring frequency groups of normal-weight participants was not statistically significant (*p*>.05) (Figure 3(b)). In summary, obesity is a factor that affects the relationship between snoring frequency and CKD prevalence.

### Multiple logistic regression analysis of the relationship between snoring frequency and CKD prevalence

At this point, we adopted different models to analyze the relevance between snoring frequency and CKD prevalence in obese and normal-weight study participants separately (Table 3). Odds ratios for snoring frequency and CKD prevalence were significant in obese participants regardless of whether we adjusted for age; sex; smoking status; drinking status; or systolic BP, TG, HDL-C, and HOMA-IR values (*p*<.05) (models 1–4). As such, snoring frequency was deemed relevant to the CKD prevalence independent of these factors in obese participants. On the contrary, the odds ratios for snoring frequency and CKD prevalence in normal-weight participants were not significant regardless of whether these factors were adjusted (*p*>.05) (models 1–4), which means that a relationship between snoring frequency and CKD prevalence does not exist in normal-weight individuals.

**Table 3.** Multiple logistic regression analysis of the association between snoring frequency and CKD prevalence.

	Never	Occasionally Odds ratio (95% CI), *p* value	Regularly Odds ratio (95% CI), *p* value
Normal-weight subjects			
Model 1	1	1.22 (0.62–2.38), .570	1.27 (0.55–2.95), .572
Model 2	1	1.18 (0.57–2.45), .657	0.84 (0.34–2.06), .703
Model 3	1	1.17 (0.56–2.43), .672	0.83 (0.34–2.04), .679
Model 4	1	1.14 (0.54–2.42), .731	0.79 (0.32–1.98), .614
Obese subjects			
Model 1	1	1.67 (0.94–2.96), .082	2.51 (1.40–4.50), .**002**
Model 2	1	1.96 (1.05–3.66), .**034**	2.55 (1.35–4.82), .**004**
Model 3	1	1.96 (1.05–3.65), .**035**	2.56 (1.35–4.86), .**004**
Model 4	1	2.03 (1.06–3.88), .**033**	2.66 (1.36–5.19), .**004**

Model 1: not adjusted; model 2: adjusted for age and sex; model 3: adjusted for age, sex, smoking status, and drinking status; model 4: adjusted as described for model 3 and SBP, TG, HDL-C, and HOMA-IR values. The bold value represents snoring frequency was deemed relevant to the CKD prevalence independent of age, sex, smoking status, drinking status, systolic BP, TG, HDL-C, and HOMA-IR values only in obese participants, but not in normal-weight participants.

## Discussion

It has been suggested that worse sleeping conditions, including shorter sleeping durations and inferior sleep quality, are associated with a higher risk for CKD in the Chinese population [20]. OSAHS is one of the manifestations of a worse sleeping quality, and CKD has been observed to develop more often in patients with OSAHS than in those without it, with the prevalence of CKD increasing with greater severity of OSAHS. On the one hand, OSAHS can directly lead to hypoxia and indirectly cause hypertension, inflammation, oxidative stress, sympathetic nervous system activation, and activation of the renin–angiotensin–aldosterone system. On the other hand, systemic hypertension can lead to glomerular hypertension and glomerular hyperfiltration, which can result in tubulointerstitial damage together with chronic hypoxia, eventually progressing to CKD [7]. However, it is worth noting that the underlying mechanisms between OSAHS and CKD mentioned above also exist in obese people [21].

It has been confirmed that snoring is a consistent assessment indicator that can reflect the severity of OSAHS, with almost 94% of OSAHS patients showing this symptom [9,13]. Moreover, the assessment of snoring is a more intuitive and convenient way to collect information in large-scale clinical studies as compared with polysomnography (PSG), which is time-consuming and complex to perform in such a setting, despite being the gold-standard modality for diagnosing OSAHS. As a result, we used snoring frequency to reflect the severity of OSAHS in the present investigation.

In this paper, we show that snoring is an independent risk factor for CKD in obese individuals. Specifically, snoring frequency was associated with CKD prevalence in our obese study participants but not in the normal-weight participants. In our study, 1845 obese participants (*n* = 1103 women) were divided into three snoring frequency groups based on their self-reported snoring frequency, which is described in Figure 1(b). We found that the CKD prevalence was statistically different between these three groups (*p*<.05) (Figure 3(a)) in obese participants and, the more frequently the obese participants snored, the greater the CKD prevalence among them – that is to say, obesity may play a role in the relationship between snoring frequency and CKD prevalence (Table 2 and Figure 3(a)). Therefore, we speculated that OSAHS patients may develop CKD under the effect of obesity.

Conversely, whether a correlation between OSAHS and CKD exists in normal-weight participants was still unclear. Therefore, we divided 1405 normal-weight participants (*n* = 938 women) into the same three snoring frequency groups (Figure 1(c)), only to find that the difference in CKD prevalence between the three groups was not statistically significant (*p*>.05) (Table 2 and Figure 3(b)). Moreover, in the multiple logistic regression analysis of the association between snoring frequency and CKD prevalence, we adjusted for factors including age; sex; smoking status; drinking status; and systolic BP, TG, HDL-C, and HOMA-IR values, which did not change the results mentioned above in either obese participants or normal-weight participants (Table 3, models 1–4). Some unadjusted factors in Table 3 such as hypoxia, inflammation, and oxidative stress were potential mechanisms of the interaction between OSAHS and CKD [7], while they hardly existed in normal-weight people. As a result, we suspected that unadjusted factors like hypoxia, oxidative stress, and inflammation may be the key factors driving CKD progression in obese OSAHS patients, which is expounded as follows.

First, the reduction in renal blood flow and increase in the oxygen consumption of renal cells lead to chronic renal tissue hypoxia, which arouses epithelial–mesenchymal transdifferentiation of the tubular cells and activates fibroblasts, resulting in damage to peritubular capillaries and renal interstitial fibrosis. Besides, renal hypoxia can reduce the oxygen tension of the renal tubulointerstitium, which can aggravate tubulointerstitial injury [22]. Second, it is reported that oxidative stress is caused by antioxidant defense mechanisms and an imbalance in the production of reactive oxygen species. OSAHS breaks the balance between reactive oxygen species removal and generation to initiate oxidative stress, which induces structural and functional damage in the kidneys [23]. Third, adipose tissue synthesizes hormones and signaling peptides known as adipokines that include leptin, adiponectin, chemerin, tumor necrosis factor-α, interleukin-6, interleukin-10, and monocyte chemoattractant protein-1, contributing to the inflammatory response in obesity-related glomerulopathy [24,25].

### Strength and limitations

To our knowledge, our study is the first to demonstrate that snoring appears to be independently associated with CKD in obese but not in normal-weight Chinese adults. This result can be used to remind those who snore frequently that they may prevent or delay the occurrence of CKD by losing weight. In this study, we used the waist circumference to evaluate the degree of abdominal obesity (central obesity). Waist circumference is a common measurement used to assess abdominal obesity, while BMI only considers an individual’s weight and height. We chose abdominal obesity as the research object given that, as compared with general obesity, abdominal obesity is correlated with a higher risk of major cardiovascular events and OSAHS [26–28]. However, there are some limitations as well. First, a cross-sectional study cannot infer causality between snoring and CKD. Second, we did not use PSG to monitor the episodes of apnea and hypopnea during sleep, which is the gold-standard modality for diagnosing OSAHS; however, PSG is not considered suitable for large-scale clinical epidemiological surveys due to its complex and time-consuming application. As compared with PSG, collecting data on snoring frequency by questionnaire is an intuitive, convenient, and consistent way to assess the severity of OSAHS [13]. Besides, snoring may be a common symptom of OSAHS and central sleep apnea (CSA) [29]. It is impossible to accurately distinguish and diagnose OSAHS and CSA without doing PSG. However, our purpose is to observe the relationship between snoring, a clinically accessible symptom, and CKD, which can at least arouse people's attention to obese snoring populations. Third, we did not conduct research on mechanisms that drive CKD progression in obese patients who snore; however, this study provides clinical evidence and clues for subsequent mechanism studies. Fourth, we did not collect and adjust the drug history and CVD events as some drugs could affect the progression of CKD and CVD events are risk factors for the prevalence of CKD [12]. For example, angiotensin-converting enzyme inhibitors (ACEIs) discontinuation increased the risk of end-stage kidney disease and subsequent death [30]. Fifth, the definition of CKD was only based on eGFR which was estimated by the CKD-EPI equation, while other kidney damage markers such as proteinuria level, urinary sediment, and albumin:creatinine ratio (ACR) were not considered [31]. Sixth, only middle-aged and elderly Chinese adults were covered in our study. The results presented above might not be appropriate for those of different ethnicities, races, or ages in China.

## Conclusions

Based on our findings, snoring appears to be independently associated with reduced eGFR <60 mL/min/1.73 m^2^ in obese but not in normal-weight Chinese adults. Further research ought to be conducted to elucidate the relevant mechanisms.

## Supplementary Material

Supplemental Material

## Abbreviations

CKD: chronic kidney disease
OSAHS: obstructive sleep apnea–hypopnea syndrome
CVDs: cardiovascular diseases
BP: Blood pressure
TG: triglyceride
HDL-C: high-density lipoprotein cholesterol
eGFR: estimated glomerular filtration rate
CKD-EPI: chronic kidney disease epidemiology collaboration
BMI: body mass index
WC: waist circumference
SBP: systolic blood pressure
DBP: diastolic blood pressure
FPG: fasting plasma glucose
HOMA-IR: homeostasis model assessment of insulin resistance
ACEIs: angiotensin-converting enzyme inhibitors
CSA: central sleep apnea
ACR: albumin:creatinine ratio
PSG: polysomnography
