# Supplementary figures and images for: Self-reported snoring is associated with chronic kidney disease in obese but not in normal-weight Chinese adults
Source: Ren Fail. 2021 Apr 26;43(1):709–17. doi: 10.1080/0886022X.2021.1915332 (PMC8079005; doi:10.1080/0886022X.2021.1915332)

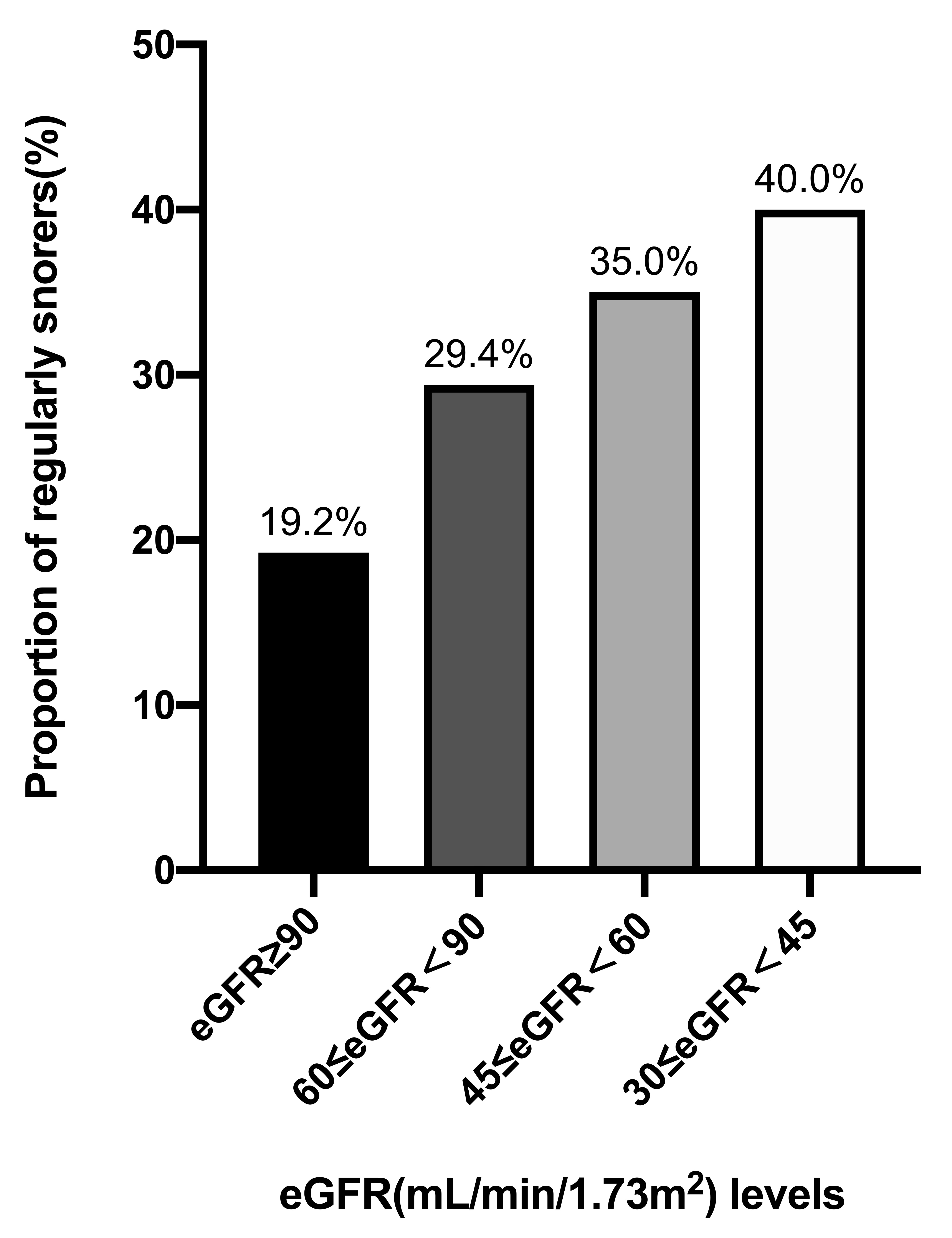

Supplement: Supplemental Material [file IRNF_A_1915332_SM8428.jpg]
